# Impact of Habitat Changes on Butterfly Diversity in the Forest–Grassland Ecotone in the Source of the Bailong River, Western China

**DOI:** 10.3390/insects17090965

**Published:** 2026-09-19

**Authors:** Min Zhao, Lei Bai, Yan Xia, Jianping He, Xiushan Li

**Affiliations:** 1 Key Laboratory of Southwest Wildlife Resources Conservation of the Ministry of Education, College of Life Science, China West Normal University, Nanchong 637009, China; zhaomin1631102@163.com (M.Z.);; 2 Center of Bailong River Forestry Protection of Gansu Province, Lanzhou 730050, China

**Keywords:** butterfly, habitat change, edge effects, vertical distribution, biodiversity conservation

## Abstract

Habitat type and elevational gradient are key drivers shaping biodiversity variation in alpine montane grasslands. To explore the effects of habitat type on butterfly communities and their variation along the elevational gradient in the forest–grassland transition zone at the source of the Bailong River, we conducted transect surveys over three consecutive years (2022–2024). The main results are as follows: (1) The butterfly community showed typical characteristics of alpine grassland assemblages, dominated by Nymphalidae (60.0%), while Lycaenidae accounted for 13.0%, followed by Pieridae (23.7%). Papilionidae and Hesperiidae accounted for very low proportions, at 0.7% and 0.5%, respectively. (2) The ecotone generated pronounced habitat edge effects; the forest-edge shrub habitat harbored the highest species richness. (3) Species-composition similarity of butterfly assemblages among habitats was low. (4) The vertical distribution of butterfly species exhibited a distinct double-valley pattern. (5) Parnassiinae species inhabiting high-elevation alpine meadows face major threats from climate change. Conservation implications: Butterfly conservation within this alpine watershed requires coordinated ecological management that balances socioeconomic development and biodiversity protection. Endemic high-elevation butterfly species should be treated as priority conservation targets for future biodiversity management in the Bailong River source area.

## Simple Summary

To explore the impacts of habitat change on butterfly communities and their variation along the elevational gradient in the forest–grassland transition zone of the Bailong River source area, we carried out transect surveys over three consecutive years (2022–2024). Our key findings are: (1) The butterfly community exhibited typical alpine grassland assemblage characteristics, dominated by Nymphalidae (60.0%), followed by Pieridae (23.7%). Papilionidae and Hesperiidae represented only small fractions, at 0.7% and 0.5%, respectively. (2) This ecotone showed pronounced habitat edge effects, and the forest-edge shrub habitat harboured the highest species richness. (3) Butterfly assemblages had low species-composition similarity across different habitats. (4) Butterfly species diversity displayed a distinct double-valley pattern in their vertical distribution. (5) Parnassiinae species living in high-elevation alpine meadows are highly threatened by climate change. They should be set as priority conservation targets for future biodiversity management in the Bailong River source area.

## 1. Introduction

Habitat loss and climate change are recognized as the primary drivers altering the distribution and abundance of flora and fauna worldwide [1,2,3]. Excessive exploitation and utilization of natural resources by humans have triggered habitat degradation and even complete loss, resulting in population declines of wild plants and animals. Forests, which support more than 80% of global wildlife, continue to disappear at a rapid rate worldwide [4]. Grassland degradation has become a critical ecological issue in China. Currently, more than 70% of China’s natural grasslands suffer from varying degrees of degradation, with moderately and severely degraded grasslands accounting for approximately 30% of the total national grassland area, severely threatening alpine biodiversity and ecosystem stability [5]. In Europe, large-scale monoculture agriculture and pesticide use have caused nearly 40% of hoverfly species, 20% of butterflies, and 9% of bees to be threatened with extinction [6]. Climate change has induced species to shift their ranges poleward and to higher altitudes, expanding their distributional ranges [3,7]. Habitat loss initially leads to a decline in population abundance, followed by a subsequent decrease in species richness [8]. While climate change exerts long-term influences on species distribution shifts, habitat degradation governs local, short-term changes in species assemblages [9]. Elevational distribution patterns of species represent one of the most intuitive manifestations of climate change. Investigating such patterns helps reveal species-specific response mechanisms to climate change and enables predictions of future range shifts and extinction risks. In parallel, exploring species composition and community structure across distinct habitats can disentangle the impacts of habitat transformation and human disturbance on biodiversity and further inform strategies for sustainable natural resource management and wildlife conservation.

As an increasing portion of the Earth’s land surface is subjected to human disturbance, many species are now on the brink of extinction. It is projected that by 2100, natural vegetation cover in biodiversity hotspots will decline by 26% to 58%. Compared to the species extinctions that have already occurred from 1500 to 2005, the number of species extinctions by 2100 is estimated to range between 220 and 21,000 (0.2% to 16%) [10]. According to the Intergovernmental Science-Policy Platform on Biodiversity and Ecosystem Services (IPBES) report [11], one million species worldwide are facing existential threats, and over the past less than 50 years, the average size of global wildlife populations has declined by 69% [10].

The impacts of climate change are extensive and profound, exerting significant effects on every dimension of biodiversity, ranging from species and populations to entire biological communities [10,12]. The influence of climate change is also evident along altitudinal gradients. As altitude increases, environmental factors such as temperature and vegetation undergo changes, which in turn have important effects on the species diversity and distribution of butterflies. Research conducted in the Gaoligong Mountains region of Yunnan demonstrates that the middle-altitude zone (1000–2000 m) harbors the highest richness of butterfly species and diversity indices, with a distinct clustering of species in low-altitude areas that are spatially separated from and rarely overlap with those in high-altitude regions [13]. It is widely acknowledged that as altitude increases, heat availability decreases, leading to a corresponding decline in species diversity [14]. Butterflies have a high demand for heat to sustain flight capabilities, promote growth and development, and adapt to diverse environmental conditions. In middle to low-altitude areas, the warm and humid climate, lush vegetation, and relatively limited human activity provide butterflies with abundant food sources and habitats, resulting in high species diversity [15]. However, as altitude further increases, climatic conditions become harsher, and vegetation types change, leading to a gradual decline in butterfly species diversity. Overall, the broad altitudinal distribution pattern and high diversity of butterflies facilitate the study of the effects of altitudinal gradient changes on species community structure.

Butterflies are widely acknowledged as reliable bioindicators for assessing ecological responses to environmental changes, due to their high sensitivity, short generation cycles, and rapid community responses to anthropogenic and climatic perturbations [1,8,16,17]. Cumulative monitoring evidence has verified that intensified land-use disturbance substantially reduces butterfly α-diversity and simplifies community structure across tropical and subtropical ecosystems, with butterfly assemblages exhibiting context-dependent responses under the combined stress of land-use alteration and climate change [18]. For instance, tropical butterfly communities are extremely vulnerable to habitat modification, as most tropical species possess strict host specificity and rely on intact forest vegetation for specialized larval host plants and adult nectar resources [19]. Moreover, pristine butterfly communities are more sensitive to climatic fluctuations than those in human-modified landscapes. Severe environmental filtering in frequently disturbed habitats excludes thermally specialized species with narrow climatic adaptability, rendering anthropogenically altered butterfly communities less responsive to climate variability [18].

As typical insect indicators for ecotone dynamics, butterflies are highly susceptible to habitat edge effects, which profoundly shape their spatial distribution, species diversity, and community assembly [19,20,21,22]. The fundamental edge effect theory proposes that transitional ecotones among forests, shrublands, and grasslands possess higher microhabitat heterogeneity, stronger light penetration, and more variable microclimatic conditions compared with homogeneous habitat interiors, supporting richer host and nectar plant resources and consequently maintaining higher insect biodiversity [23,24]. Consistent with this theory, ecosystem boundaries consistently harbor higher butterfly species richness than single, uniform habitats, because transitional zones integrate vegetation and resource characteristics of adjacent ecosystems, simultaneously accommodating forest-dependent specialists and open-habitat generalists [19,25]. Similar diversity-promoting edge effects have been validated in farmland–woodland ecotones, where woodland edges provide shelter and overwintering microhabitats while adjacent farmlands supply seasonal floral resources, jointly sustaining richer pollinator assemblages than isolated single habitats [25,26]. For butterflies specifically, edge zones serve as critical corridors for foraging, dispersal, and habitat colonization, acting as core biodiversity hotspots in mountainous and fragmented landscapes [26].

Current empirical studies have formed a unified consensus on butterfly edge effects in mountain and plateau ecosystems. Positive edge effects dominate forest–grassland transitional landscapes, where ecotones exhibit higher butterfly species richness, individual abundance, and community diversity than closed forest interiors and pure grasslands [26,27]. Additionally, butterfly diversity presents a distinct spatial gradient along edge distance, with the 0–200 m boundary range functioning as the optimal distribution zone for butterfly communities [28].

Despite the abundant research on butterfly elevational patterns and edge effects globally, most existing studies focus on tropical, subtropical, and low-altitude temperate mountain regions, while systematic research on alpine butterfly community assembly and disturbance responses on the eastern Qinghai–Tibet Plateau remains insufficient. The Bailong River source area, located in the transitional zone of the western Qinling Mountains and the eastern Qinghai–Tibet Plateau, features fragile alpine ecosystems and complex forest–grassland ecotones, providing an ideal natural platform to explore the combined effects of habitat heterogeneity and anthropogenic disturbance on butterfly communities. However, the vertical distribution rules, species similarity patterns, and driving mechanisms of butterfly communities in this watershed remain unclear, restricting targeted biodiversity conservation and ecological restoration in alpine watershed ecosystems.

This study aims to elucidate the impacts of different habitat types on butterfly community structure and to investigate the distribution patterns of butterfly communities along altitudinal gradients in the source area of the Bailong River. The results will furnish additional scientific evidence to bolster conservation and restoration initiatives, focused on executing proactive and efficient strategies to tackle the ecological issues arising from climate change and anthropogenic actions. This will ultimately foster the sustained advancement of the environment in the Diebu–Ruo’ergai County area.

Against this research background, this study proposes three core hypotheses regarding butterfly community distribution patterns: (1) The species composition exhibits typical characteristics of alpine grasslands, with Nymphalidae and Pieridae being the most abundant families, while Papilionidae and Hesperiidae are the least abundant; (2) among the three habitat types, the forest–grassland ecotone supports the highest level of butterfly diversity; and (3) butterfly species richness exhibits a monotonically decreasing trend with increasing altitude.

## 2. Materials and Methods

### 2.1. Study Area

The source area of the Bailong River is situated at the junction of Diebu County (Gannan Tibetan Autonomous Prefecture, Gansu Province) and Ruo’ergai County (Aba Tibetan and Qiang Autonomous Prefecture, Sichuan Province), covering the geographic coordinates of 102°08′–103°39′ E and 33°06′–34°20′ N. The entire study area spans approximately 12,000 km^2^, with an elevation gradient ranging from 2200 m to 4920 m. As a key zone on the northeastern margin of the Qinghai–Tibet Plateau, this region features rugged terrain, magnificent mountain and river landforms, diverse natural landscapes, and abundant natural resources. The local vegetation is primarily composed of primary coniferous forests and alpine grasslands. As an important tributary of the upper Yangtze River, the Bailong River originates in the vicinity of Langmusi Temple. The regional landscape is dominated by extensive primary forests and alpine grasslands, with scattered and limited agricultural lands, forming a unique and integrated agro-forestry–pastoral composite ecosystem with high ecological value. Functioning as a crucial water conservation zone and core ecological barrier, this region plays an irreplaceable role in maintaining the ecological security of the upper Yangtze River basin. Spatially complex terrain restricts the construction of transport infrastructure here, resulting in underdeveloped local economic conditions and a single industrial structure dominated by livestock breeding. Historically, forest logging and grazing have long been the main economic pillars of the region. Long-term unreasonable human activities have triggered a series of ecological problems, including decreased forest cover, degraded ecosystem functions, widespread grassland degradation, severe soil erosion, and continuous biodiversity loss [29].

### 2.2. Habitat Types and Transect Selection

**Figure 1:**
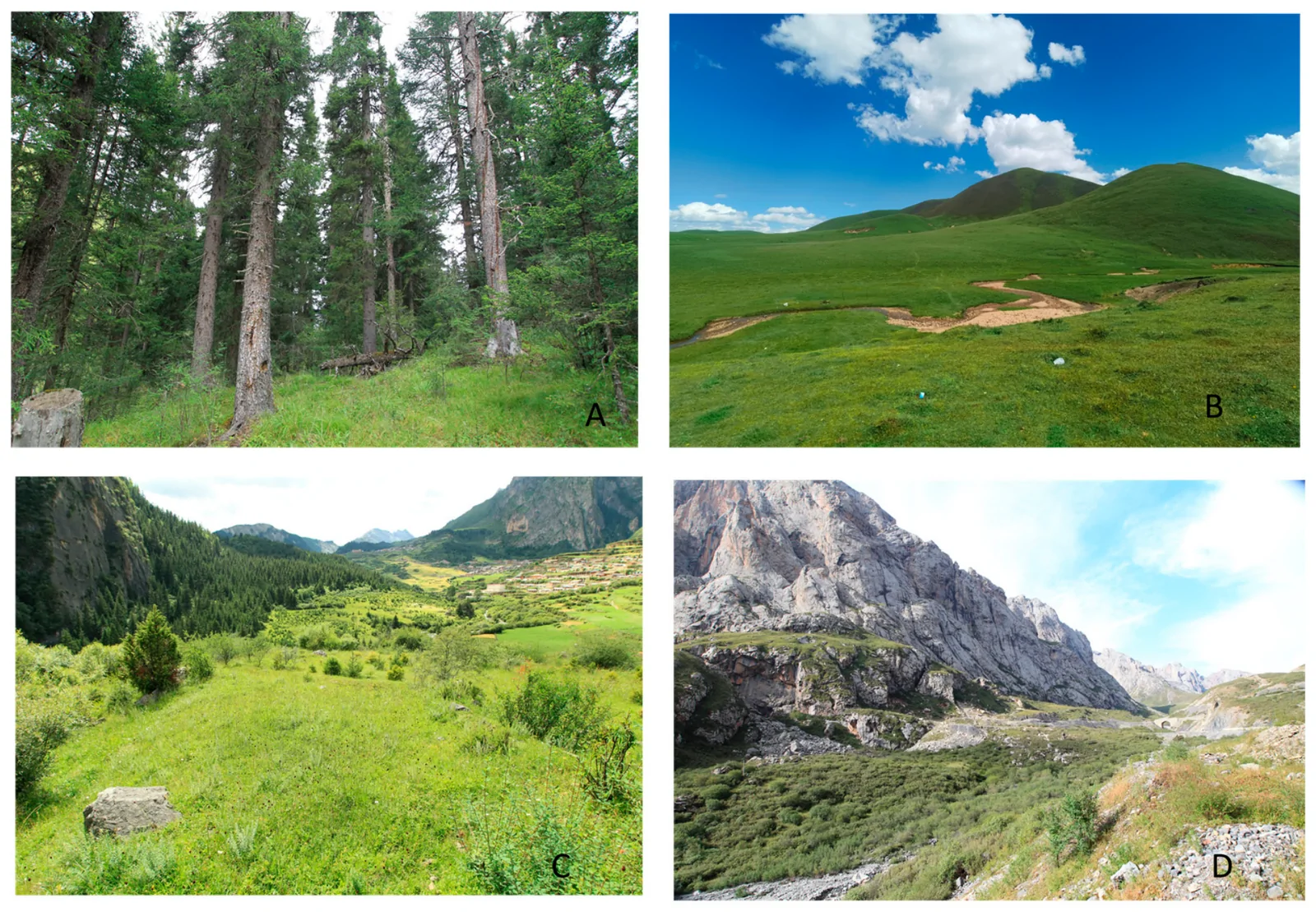
Types of habitats inhabited by butterflies in the source region of the Bailong River. (**A**) Coniferous forest; (**B**) large-scale grassland; (**C**) shrubs and grassland on the edges of forest; (**D**) alpine meadow.

The headwaters of the Bailong River are located in a forest–steppe ecotone with typical alpine gorge terrain, showing distinct vertical vegetation zonation. The forest zone at elevations of 2100–2900 m is dominated by mixed coniferous–broadleaved forests, while extensive subalpine dark coniferous forests prevail at 2900–3600 m. These high-altitude coniferous forests are mainly composed of *Abies faxoniana* and *Abies fargesii*, characterized by high canopy closure and low tree species diversity, providing inferior habitats for butterflies. Grassland habitats distributed at 3000–3600 m have relatively simple community structures, predominantly consisting of herbaceous vegetation such as *Carex muliensis* and *Caltha palustris*. Vegetation shifts to alpine shrub–meadow above 3600 m [29]. Forest edge shrub habitats, covered with scattered shrubs, small trees and ground herbs, serve as crucial transitional ecotones between forest and grassland ecosystems. Agricultural lands in this region are restricted to gentle valley bottoms, where small-scale croplands are cultivated with highland barley, rapeseed, and wheat, accounting for only a minor proportion of the total landscape [29]. Due to the poor suitability of dense interior forests for butterfly survival, local butterfly species predominantly inhabit forest edges, alpine grasslands, and mountain meadows. Accordingly, these three typical habitat types were selected as the field survey sites in this study (Figure 1).

**Figure 2:**
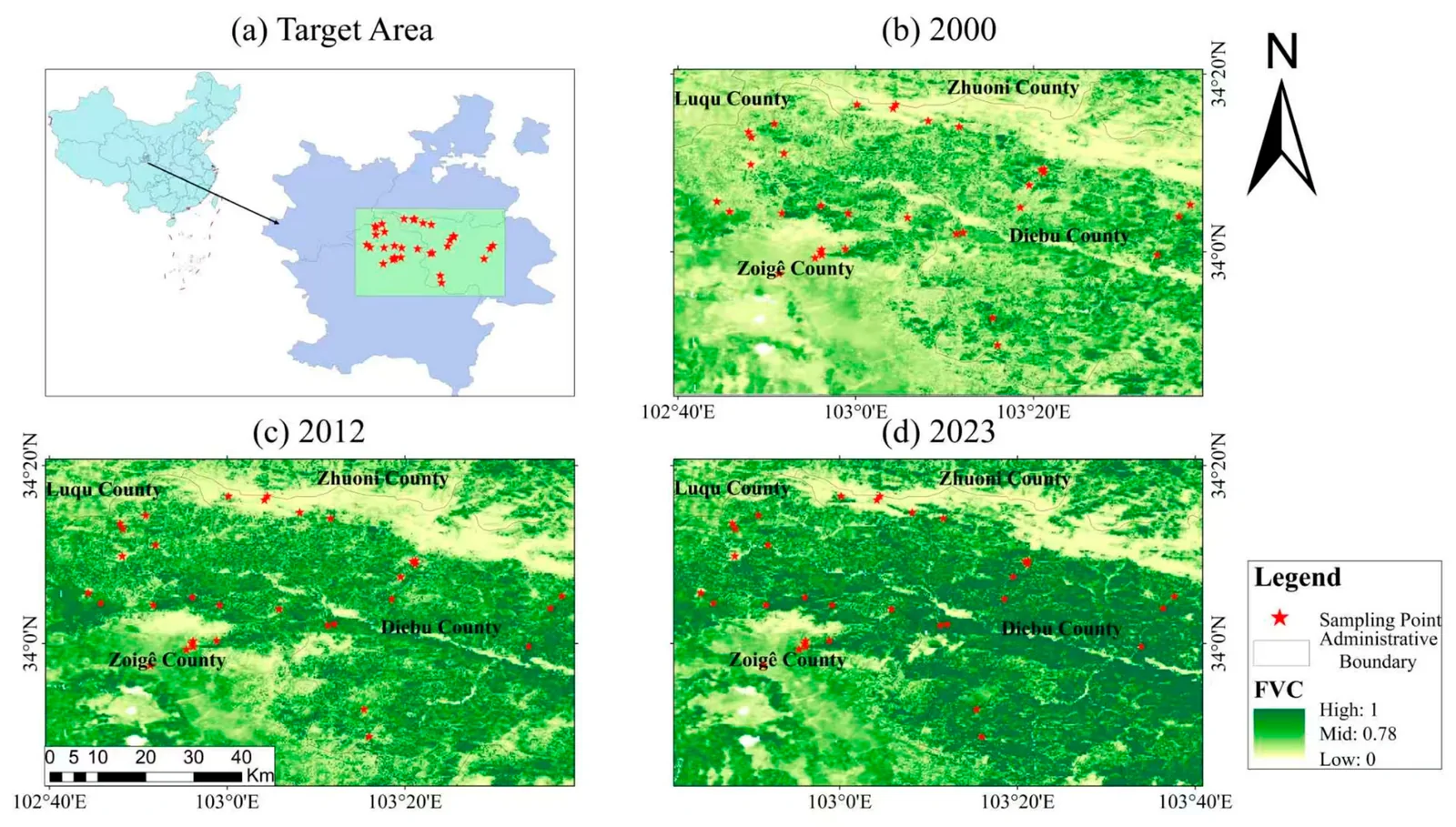
Butterfly survey sites and changes in forest vegetation coverage (FVC) in the headwaters of the Bailong River (2000: 73.86%, 2012: 78.93%, 2023: 81.54%).

Since the Chinese government launched the Natural Forest Protection Program and implemented a comprehensive ban on natural forest logging in 1998, the local forest ecosystem has achieved remarkable restoration. The regional forest coverage has increased steadily, and the ecological functions of forests in soil and water conservation and biodiversity conservation have gradually recovered (Figure 2). Nevertheless, the study area still faces prominent ecological disturbances in recent years. Persistent overgrazing and intensifying tourism development pressure, together with road construction activities, have caused severe damage to forest edge shrublands and native grasslands. Widespread grassland degradation induced by long-term overgrazing has further intensified the contradiction between regional socioeconomic development and ecological protection.

Transect selection in this study comprehensively considered traffic accessibility, complete altitudinal gradient coverage, and representative habitat types, ensuring that at least one transect was set for every 200 m elevation interval. More than 40 fixed transects with a uniform length of 200 m were initially established in 2022. However, field disturbances, including habitat damage caused by tourism development and traffic interruption induced by seasonal floods, destroyed several sampling routes during the consecutive surveys from 2023 to 2024. Finally, a total of 34 intact and valid transects were retained across the accessible elevation range of 2200–4200 m (Figure 2). Specifically, 6 transects were set in grassland habitats, 10 in alpine meadows, and 18 in forest edge shrub habitats. Considering the uneven transect distribution across the three habitat types, a balanced dataset consisting of 6 transects from each habitat (18 transects in total) was further screened for subsequent comparative analysis. For altitudinal gradient pattern analysis, we selected transects along a river valley from valley bottom to mountain top; the entire 2200–4200 m elevation range was divided into ten 200 m vertical bands, with valid data from 16 standardized transects adopted for subsequent statistical analysis, guaranteeing effective sample coverage for each elevation interval.

### 2.3. Transect Survey

Field surveys were conducted annually four times from May to August during 2022–2024. Prior to each survey, environmental variables along each transect were measured and recorded using a thermos-hygrometer, handheld anemometer, and GPS locator. The recorded environmental indicators included altitude, latitude, longitude, real-time air temperature, relative humidity, and wind speed. In addition, vegetation-related parameters such as forage grass height and the species richness and density of nectar plants were systematically investigated. See Supporting Information SA: Environmental factors, and transect counting results.

Butterfly surveys were performed following Pollard’s standard transect method [30]. All field investigations were conducted on sunny, windless days when ambient temperature exceeded 14 °C. Surveyors walked slowly along each predefined transect at a constant speed of 2.5 km/h, recording and sampling butterfly individuals within a vertical height of 5 m and a horizontal width of 2.5 m on both sides of the transect. Readily identifiable butterfly species were recorded directly in the field, whereas unknown specimens were collected with insect nets, placed temporarily in triangular paper envelopes, and brought back to the laboratory for morphological identification. For every specimen, we carefully recorded metadata including sampling locality, sampling date, and collector information. All specimens are preserved in the laboratory of the College of Life Science, China West Normal University. The full transect dataset is provided in Supporting Information SB: transects counting data.

Butterfly species identification was conducted based on authoritative taxonomic monographs, including <Illustrations of Chinese Butterflies> and <Ecological Illustrated Handbook of Chinese Insects>.

### 2.4. Data Analysis

#### 2.4.1. Biodiversity Index Analysis

Four diversity indices were selected to reflect the diversity of the butterfly community, namely, Margalef’s richness index (*R*), the Shannon–Wiener index (*H′*), the Pielou evenness index (*E*), and Simpson’s dominance index (*D*) [31,32]. The calculation formulas are as follows:

The Margalef species richness index (*R*) is employed to quantify the species richness within a community:

(1) $$R=\left(S-1\right)/\ln N$$

The Shannon–Wiener diversity index (*H’*) represents the species richness and evenness of a community:

(2) $$H^{\prime }=-\sum Pi\ln Pi,\;Pi=\frac{Ni}{N}$$

The Pielou evenness index (*E*) represents the degree of evenness in the distribution of individual numbers among different species:

(3) $$E=\frac{H^{\prime }}{lnS}$$

The Simpson dominance index (*D*) is used to measure the degree of concentration and dominance within a community. This index ranges from 0 to 1, with higher values indicating greater community diversity:

(4) $$D=1-\sum {Pi}^{2}$$

where *S* represents the total number of butterfly species; *N* is the total number of individuals across all species; *Ni* indicates the number of individuals of species *i*; *Pi* denotes the proportion of individuals of species *i* to the total number of individuals; species with *D* ≥ 25% are classified as extremely dominant species; those with *D* ranging from 5% to 25% (excluding 25%) are considered dominant species; species with *D* between 0.5% and 5% (inclusive of 0.5%) are regarded as common species; those with *D* between 0.1% and 0.5% are classified as rare species; and species with *D* less than 0.1% are deemed extremely rare species [33].

#### 2.4.2. ANOSIM and NMDS Analysis

To compare inter-species differences across habitat types, we selected six transects in each of the three habitats (only six transects remained for grassland due to tourism-related development). Non-parametric two-factor analysis of similarities (ANOSIM) and non-metric multidimensional scaling (NMDS) based on Bray–Curtis dissimilarity were employed to compare and analyze the differences in butterfly communities across different habitats. The ANOSIM results were judged based on the R value, which reflects the degree of difference between and within groups, ranging from (−1, 1). An R > 0 indicates the presence of inter-group differences, with values closer to 1 indicating greater differences; an R < 0 suggests that inter-group differences are smaller than intra-group differences, rendering the grouping insignificant. The NMDS results were evaluated using the stress coefficient, with a stress < 0.05 indicating excellent results; 0.05 < stress < 0.1 suggesting very good representation; 0.1 < stress < 0.2 indicating that the NMDS analysis has some reliability and the two-dimensional plot has some explanatory significance; and a stress > 0.2 rendering the analysis results uninterpretable [34].

#### 2.4.3. ANOVA Analysis

To better characterize butterfly distributions along the elevational gradient and associated disturbance factors, we established eight elevational bands across a continuous river valley spanning an elevational difference of 1600 m from low-elevation sites to mountain summits. Each elevational band covered 200 m and contained two transects and a total of 16 transects. SPSS 22.0 was employed to conduct one-way ANOVA on the butterfly community composition, species diversity, and nectar plant density index across different habitat types and altitudes; the Least Significant Difference (LSD) method was utilized to assess the significance of differences between groups.

#### 2.4.4. PERMANOVA Analysis

To comprehensively evaluate the effects of elevation and habitat type on butterfly abundance and species richness, multivariate permutational analysis of variance (PERMANOVA) was applied. Data from all 34 transects were used for the analysis. A multivariate response matrix was constructed based on butterfly abundance and species richness for each transect. Given the large discrepancy in value ranges between the two metrics and the strongly skewed distribution of abundance, abundance and species richness were log(x + 1)-transformed prior to analysis and subsequently standardized to mitigate the influences of extreme values and dimensional differences on analytical outputs. Euclidean distance was computed from the standardized response matrix.

Elevation was treated as a continuous variable, whereas habitat type was treated as a categorical variable comprising three categories: grassland, alpine meadow, and forest-shrubland. A full model containing elevation, habitat type, and their interaction was first fitted to test whether elevation effects varied across habitat types. If the interaction term was non-significant, an additive model retaining only the main effects of elevation and habitat type was adopted. Marginal permutation tests were then performed to assess the independent effects of elevation and habitat type after controlling for the other factor. Significance testing in PERMANOVA was implemented with 9999 random permutations.

Permutational multivariate dispersion test (PERMDISP) was additionally used to examine homogeneity of within-group dispersion among habitat types, so as to determine whether habitat differences detected by PERMANOVA could be confounded by heterogeneous within-group dispersion. When the overall effect of habitat type was significant, pairwise PERMANOVA comparisons among habitat types were conducted, and Bonferroni correction was applied to *p*-values for multiple comparisons. The significance threshold was set at *p* < 0.05.

#### 2.4.5. Bray–Curtis Dissimilarity Coefficient Analysis

The Bray–Curtis dissimilarity coefficient (distance index) was used to compare and cluster the differences and similarities in butterfly communities across different altitudes. The specific formulas are as follows:

(5) $$D_{Bray-Curtis}=1-\frac{\sum min\left(S_{A,i},S_{B,i}\right)}{\sum S_{A,i}+\sum S_{B,i}}$$

In the formula, let $S_{A,i}$ represent the richness of species i in community *A* and $S_{B,i}$ represent the richness of species i in community *B*. The value ranges from 0 to 1: when the value is 0, it means there is no difference between the two communities; the closer the value is to 1, the greater the difference between the two communities. When the value falls within the range of 0.75 to 1, it indicates that the two communities are very dissimilar; when the value is between 0.5 and 0.75, it suggests that the two communities are moderately dissimilar; when the value ranges from 0.25 to 0.5, it shows that the two communities are moderately similar; and when the value lies within the range of 0 to 0.25, it implies that the two communities are extremely similar.

Correlation between butterfly diversity and nectar-source plants across different habitat types was analyzed by means of Spearman correlation analysis.

## 3. Results

### 3.1. Community Composition

A total of 9295 butterflies, comprising 134 species belonging to 5 families and 69 genera, were collected and recorded (Table 1).

### 3.2. Indices of Butterfly Diversity Across Three Habitat Types

The species counts, individual counts, and diversity indices in the three habitat types are presented in Table 2.

The species richness, abundance, Margalef index, Shannon–Wiener index, and Simpson index were all highest in the forest-edge shrub habitat, significantly surpassing those of the butterfly communities in the grassland and alpine meadow habitats, while the Simpson’s dominance index was high but not significantly so. In contrast, the Pielou index was highest in the alpine meadow, indicating that individuals are distributed more evenly among different species within the alpine meadow community, and no single or few species overwhelmingly dominate the assemblage.

### 3.3. Similarity of Butterfly Community Structure Across Three Habitat Types

**Figure 3:**
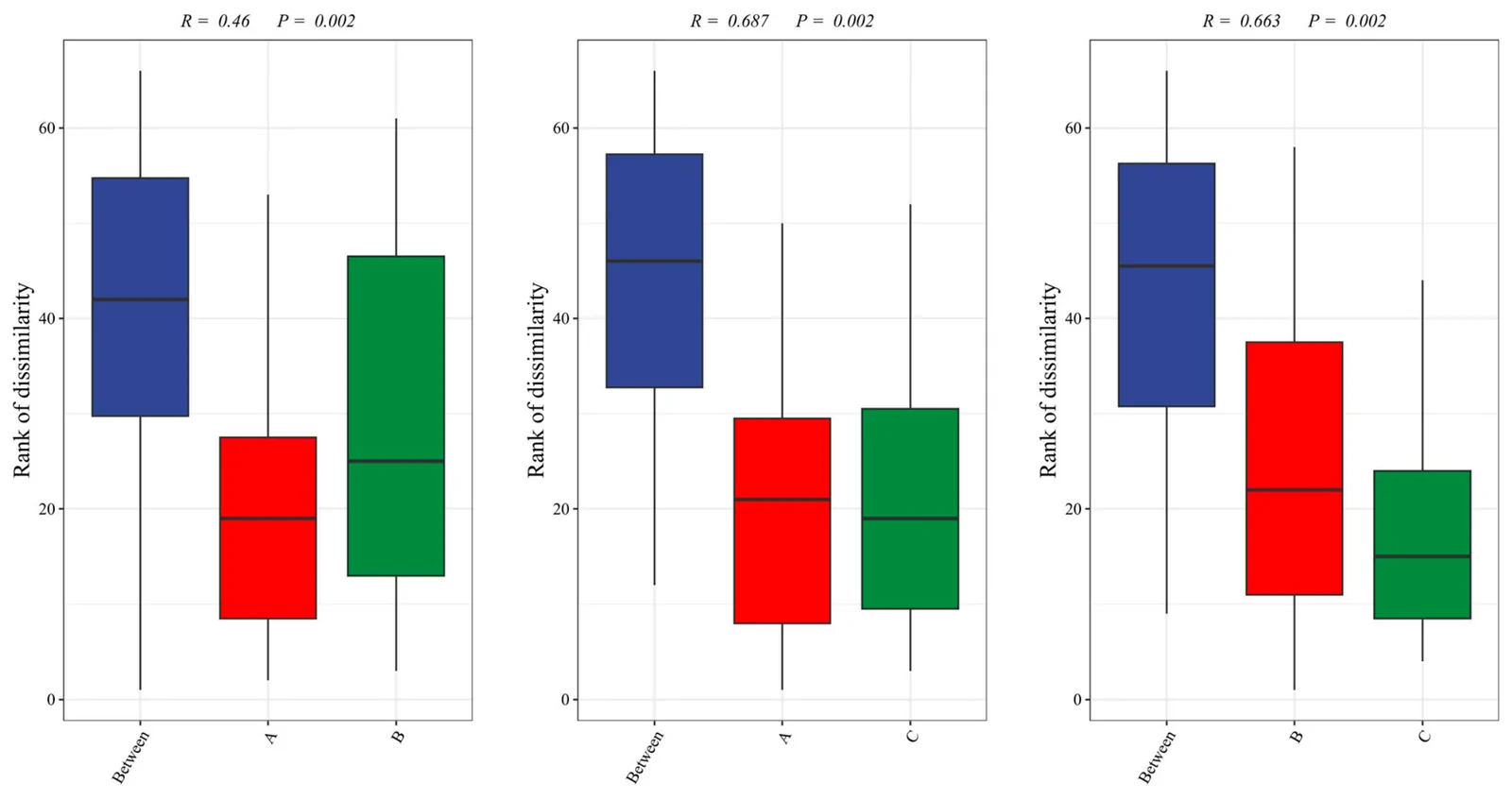
Analysis of the similarity of butterfly communities in different habitats in the source region of the Bailong River. (A) Grassland; (B) alpine meadow; (C) forest–shrubland.

According to the results of the ANOSIM analysis, the between-group difference values between the grassland and alpine meadow habitats were higher than the within-group values for these two habitat types (R = 0.46), indicating a highly significant difference (*p* = 0.002). Similarly, the between-group difference values between the grassland and forest-edge shrub habitats exceeded the within-group values for these two types (R = 0.687), also showing a highly significant difference (*p* = 0.002). The between-group difference between the alpine meadow and forest-edge shrub habitats was also higher than their respective within-group values (R = 0.663), with a highly significant difference (*p* = 0.002) (Figure 3). These findings suggest that the species compositions among the three habitat types are highly dissimilar.

**Figure 4:**
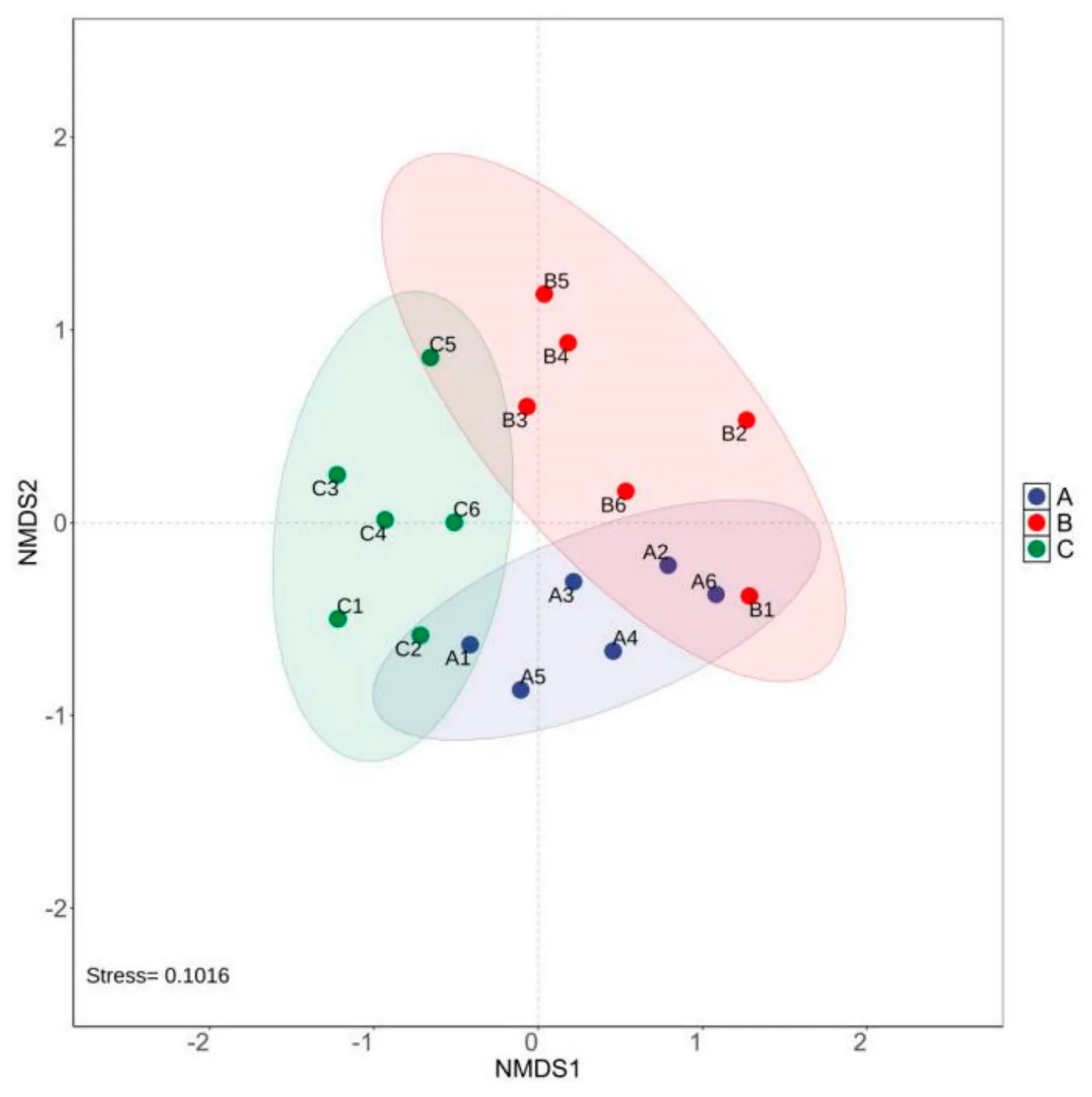
Non-metric multidimensional analysis of butterfly communities in the source region of the Bailong River. (A) Grassland; (B) alpine meadow; (C) forest-edge shrubs.

The non-metric multidimensional scaling (NMDS) stress coefficient for butterfly communities across different habitats was 0.1016, approaching 0.1. As depicted in Figure 4, butterfly communities in the three habitat types exhibited relatively clustered distributions within their respective habitats. While there were intersecting sections among the habitat types, the degree of overlap in species composition was low, and they were relatively separated on the NMDS ordination plot. Therefore, the similarity in butterfly community composition among the three habitat types is low, and they can be classified into three distinct categories (Figure 4).

### 3.4. Vertical Distribution Pattern of Butterfly Communities

**Figure 5:**
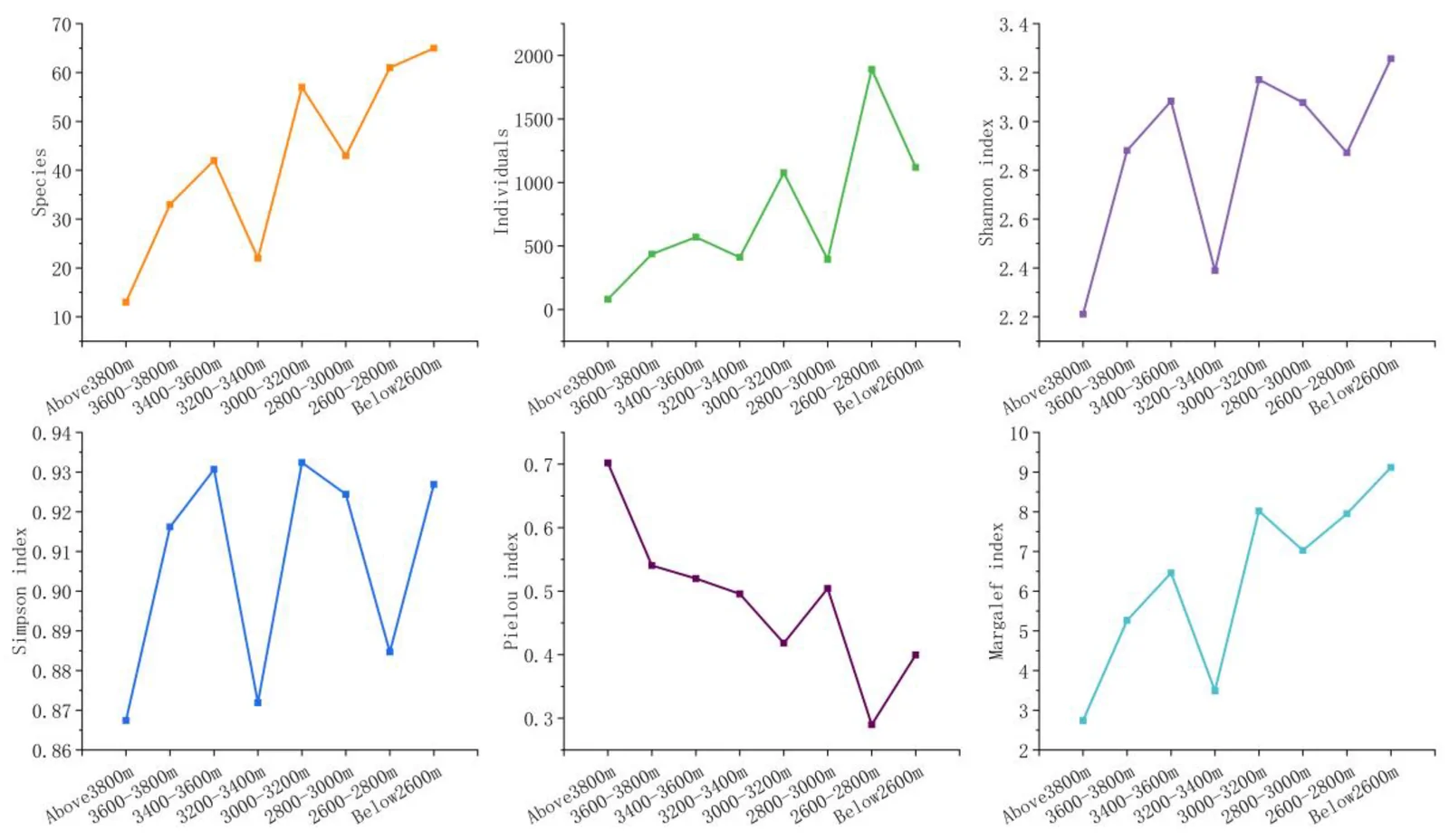
Changes in butterfly diversity at different altitudes in the source region of the Bailong River.

ANOVA results showed that species richness, abundance, the Shannon–Wiener diversity index, the Simpson’s dominance index, and the Margalef richness index decreased gradually with increasing elevation. However, these metrics exhibited a double-valley pattern, with significant declines occurring at 2800–3000 m and 3200–3400 m. These elevation ranges correspond to areas subject to intensive tourism and overgrazing. In contrast, the Pielou evenness index increased gradually with rising elevation (Figure 5).

### 3.5. General Distribution Pattern in Altitude and Habitat Gradient

**Figure 6:**
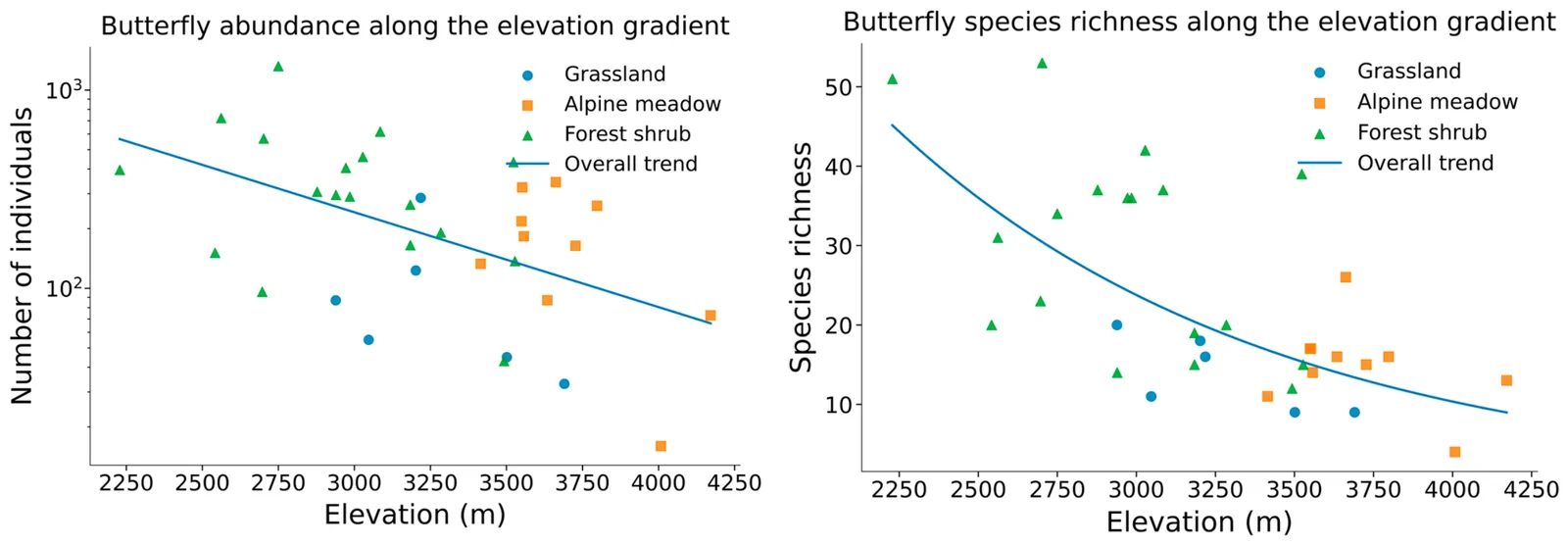
Variation in butterfly abundance and species richness along the elevational gradient. Different symbols represent the three habitat types: grassland, alpine meadow, and forest–shrubland. Scatter points denote the raw observed values from each survey transect, and curves indicate the overall trends along elevation. The *y*-axis for the abundance panel is log-scaled to visualize variations across transects with disparate magnitudes of counts.

Results from multivariate PERMANOVA revealed a non-significant interaction between elevation and habitat type (pseudo-F = 0.448, *R*^2^ = 0.016, *p* = 0.703), indicating no evidence that the combined effects of elevation on butterfly abundance and species richness varied substantially across habitat types. Accordingly, an additive model containing only the main effects of elevation and habitat type was further adopted for subsequent analyses (Figure 6).

The additive model was statistically significant overall (pseudo-F = 9.187, *R*^2^ = 0.479, *p* < 0.001), demonstrating that elevation and habitat type together exerted significant influences on the joint variation in butterfly abundance and species richness. This full additive model explained approximately 47.9% of the multivariate variation.

After accounting for the effect of habitat type, elevation remained statistically significant (pseudo-F = 8.084, *R*^2^ = 0.140, *p* = 0.006), independently explaining around 14.0% of the multivariate variation. Combined with transect observations, both butterfly abundance and species richness generally decreased with increasing elevation, suggesting that the elevational gradient constitutes a key driver shaping variation in butterfly abundance and species richness within the study area.

**Figure 7:**
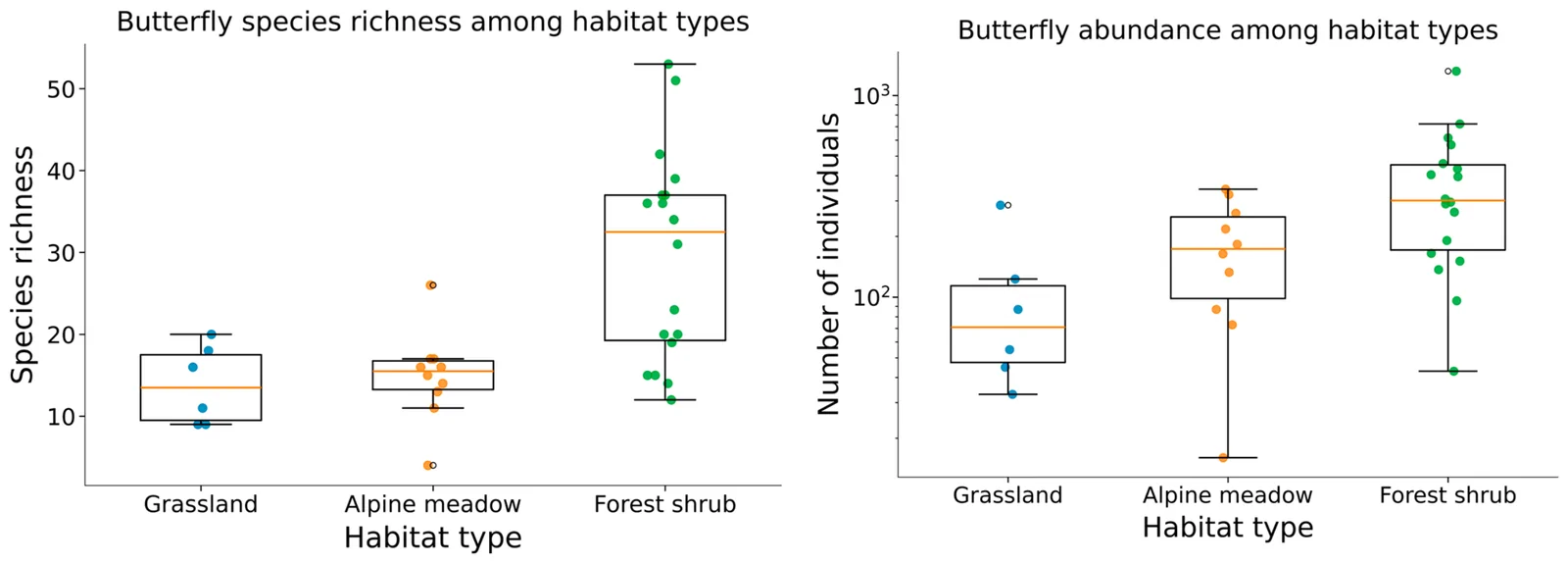
Differences in butterfly abundance and species richness across habitat types. Box-plots illustrate the data distribution for each habitat, and scatter points represent raw observed values from individual survey transects. The overall effect across the three habitat types was significant; following Bonferroni correction, only the pairwise difference between forest-shrubland and grassland was statistically significant.

After accounting for elevation, habitat type also exerted a significant effect (pseudo-*F* = 3.874, *R*^2^ = 0.135, *p* = 0.021), independently explaining approximately 13.5% of the multivariate variation. Among habitat types, forest–shrubland generally supported higher butterfly abundance and species richness, grassland showed the lowest values, and alpine meadow was intermediate between the two. The permutational multivariate dispersion test revealed no significant difference in within-group dispersion across habitat types (PERMDISP, *p* > 0.05), suggesting that the habitat-related differences detected via PERMANOVA were unlikely to be primarily driven by heterogeneous data dispersion within habitats (Figure 7).

Given the significant overall effect of habitat type, pairwise comparisons among habitats were subsequently performed. Following Bonferroni correction, the difference between forest–shrubland and grassland was significant (adjusted-*p* = 0.030). By contrast, comparisons between alpine meadow and grassland (adjusted-*p* = 0.098), and between alpine meadow and forest–shrubland (adjusted-*p* = 1.000), were non-significant.

Collectively, both elevation and habitat type significantly shaped butterfly abundance and species richness in the study area, with comparable amounts of independent explanatory power. Elevation independently explained around 14.0% of the variation, whereas habitat type explained approximately 13.5%. This indicates that the spatial patterns of butterfly abundance and species richness in the study region are not determined by a single factor but are jointly modulated by elevational gradients and habitat conditions.

### 3.6. Similarity in the Composition of Butterfly Community Structure Across Different Altitude Gradients

**Figure 8:**
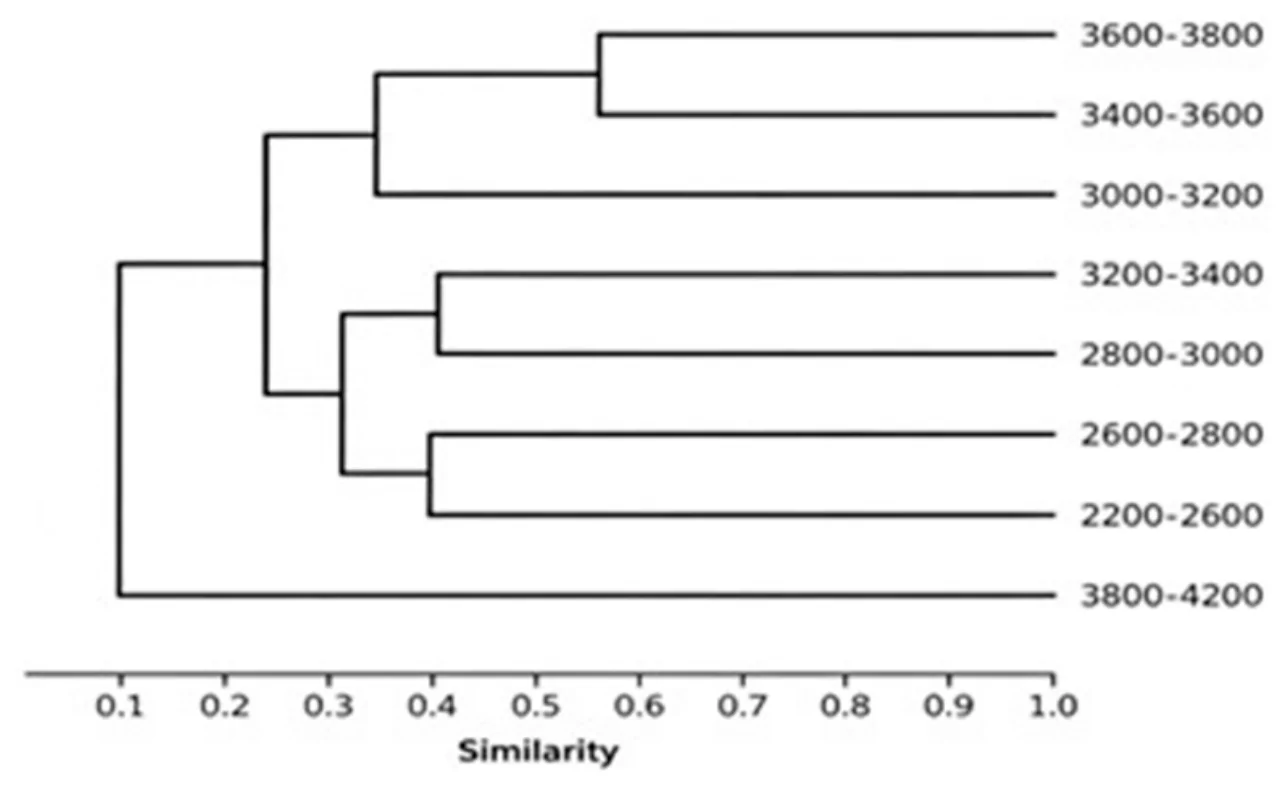
Similarity clustering of butterfly communities at different altitudes in the source region of the Bailong River.

Based on the calculation of the Bray–Curtis dissimilarity index for species composition across different altitudes, the similarity index between the high-altitude range above 3800 m and other altitude ranges is approximately 0.1 (ranging from 0.04 to 0.19), with dissimilarity coefficients all above 0.75. This indicates that the butterfly communities above 3800 m are highly dissimilar to those at other altitudes. The similarity indices for butterfly communities between 3000–3200 m and 3400–3600 m, as well as between 3200–3400 m and 2600–2800 m, fall within the range of 0.2 to 0.4, with dissimilarity coefficients between 0.5 and 0.75, suggesting relatively low similarity among these communities. The butterfly communities at 3400–3600 m and 3600–3800 m exhibit the highest vertical distribution similarity, with a similarity index of 0.5606, and are clustered together at a dissimilarity coefficient of 0.4394. At a dissimilarity coefficient of 0.5950, the altitude ranges of 3200–3400 m and 2800–3000 m are grouped together, with a similarity index of 0.4050. The altitude ranges of 2600–2800 m and 2200–2600 m are clustered at a dissimilarity coefficient of 0.6044, with a similarity index of 0.3956 (Figure 8).

### 3.7. Correlation Between Butterfly Diversity and Nectar-Source Plants Across Different Habitat Types

**Figure 9:**
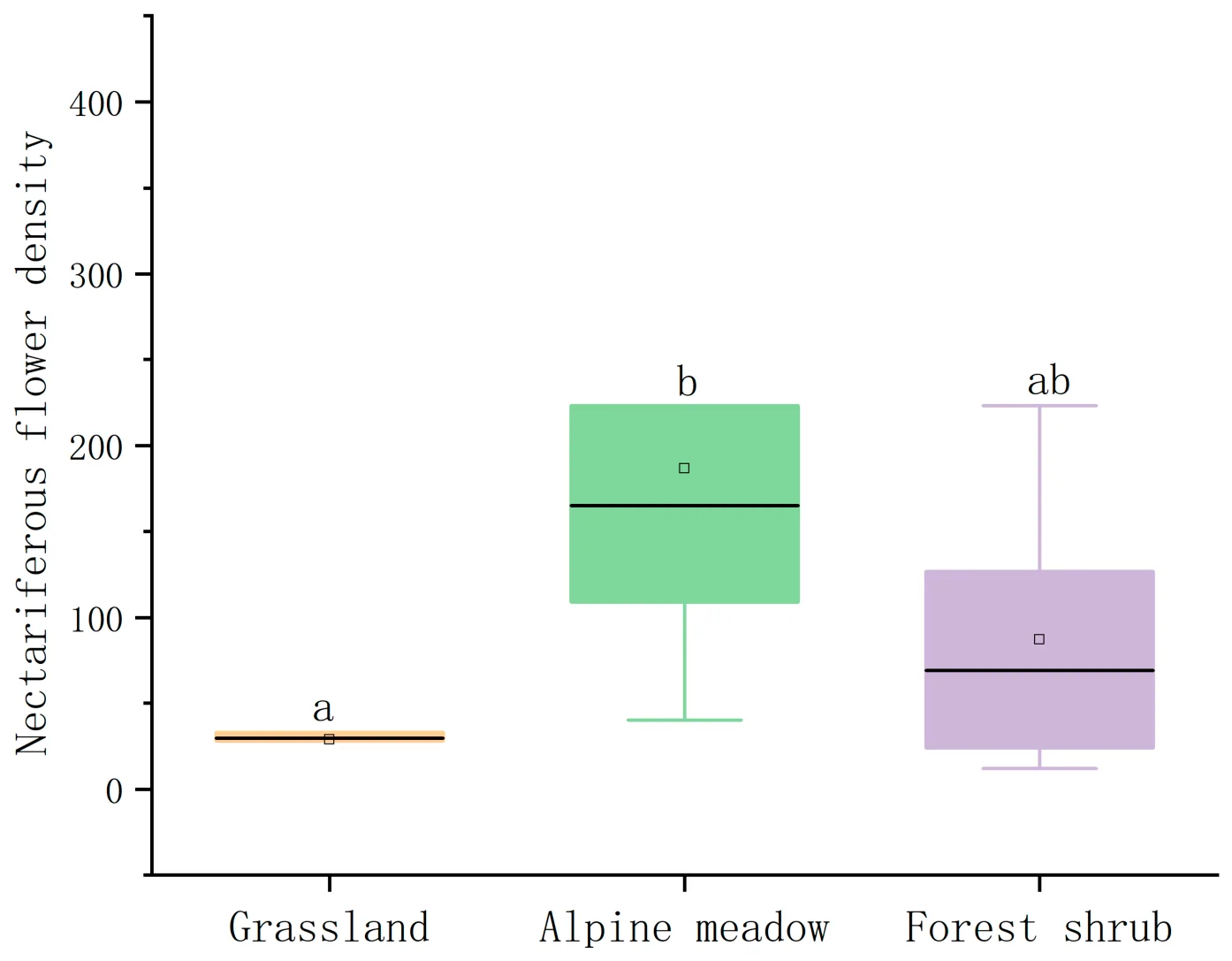
Differences in the density of nectariferous flowers in different habitats in the source region of the Bailong River. (a. density of nectar plant in grassland, b. density of nectar plant in alpin meadow, ab. density of nectar plant in forest shtub).

There are significant differences in the density of nectar-source plants across different habitat types. The alpine meadow habitat exhibits the highest floral richness and density, followed by forest-edge shrub habitats, while grassland habitats have the lowest floral density, showing a marked difference from the alpine meadow habitat (Figure 9).

**Figure 10:**
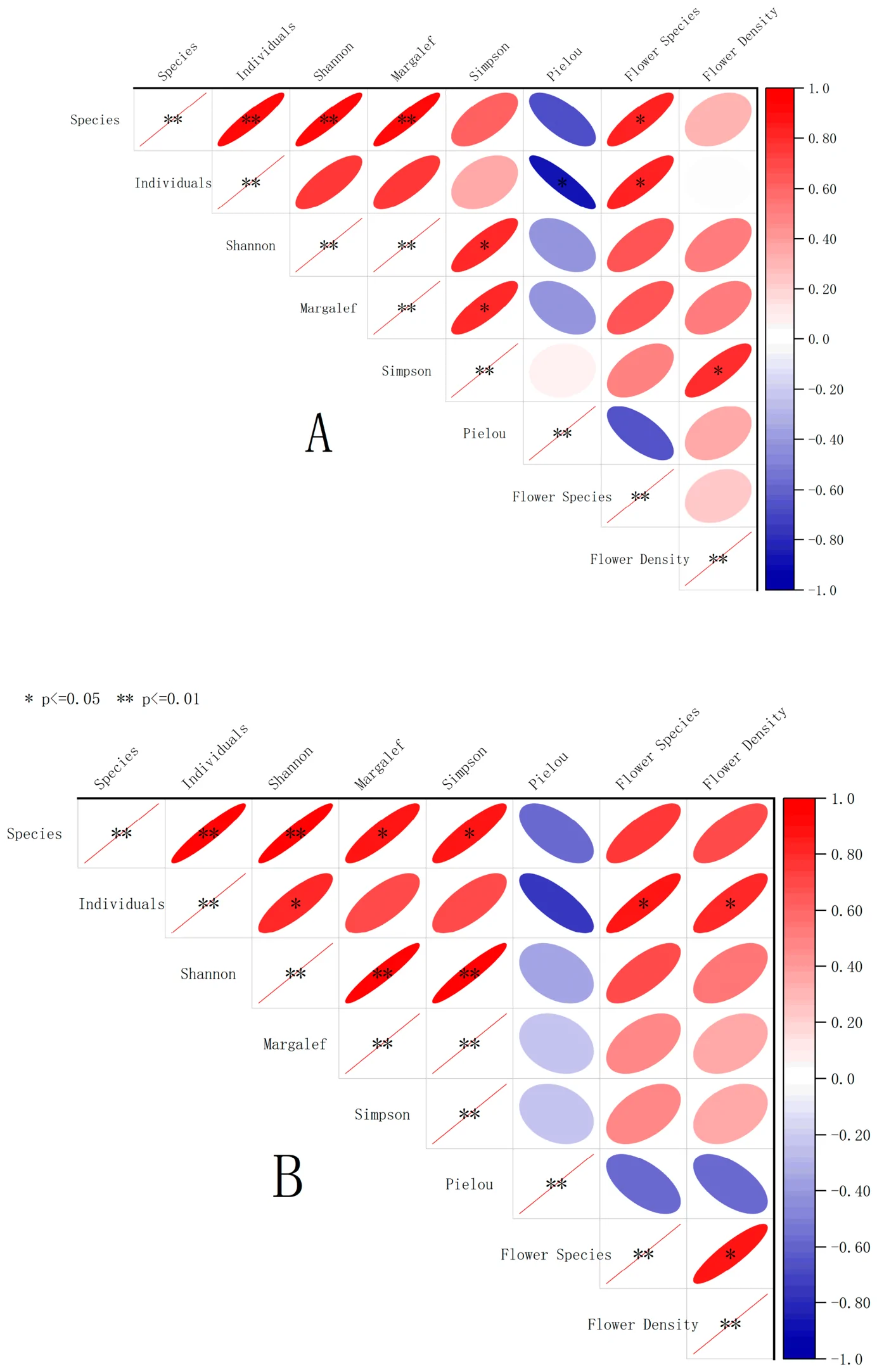

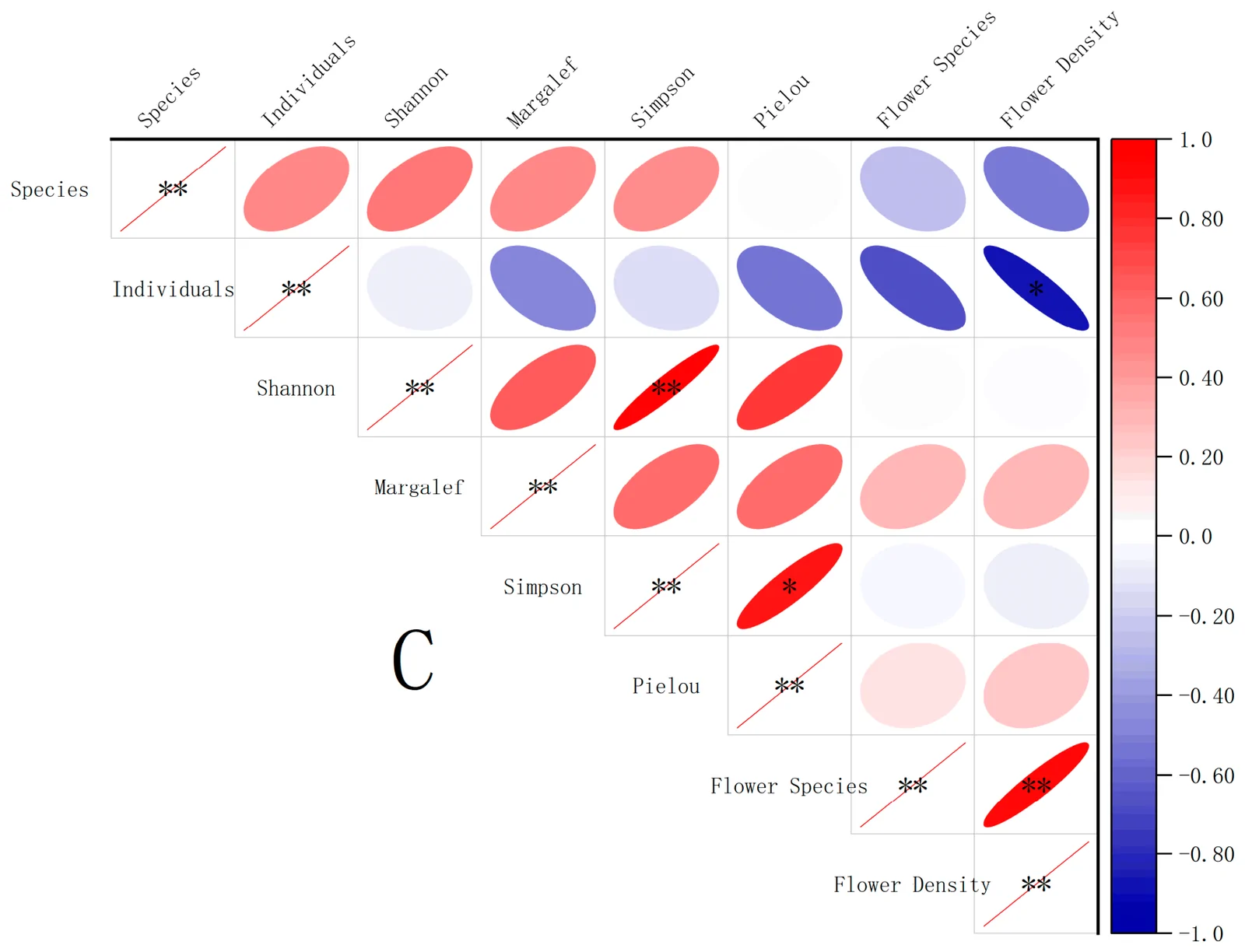
Heat map of the correlation between butterfly community diversity and nectariferous plants in the source region of the Bailong River. (**A**) Grassland; (**B**) alpine meadow; (**C**) forest–shrubland. Note: “*” denotes a statistically significant correlation at the 0.05 significance level (two-tailed test), while “**” indicates a highly statistically significant correlation at the 0.01 significance level (two-tailed test).

According to the results of Spearman correlation analysis, in the alpine meadow habitat, there is a significant positive correlation between the species richness of butterfly communities and the species richness of nectar-source plants. Notably, butterfly abundance shows a highly significant positive correlation with the variety and density of flowers (correlation coefficients: 0.886 and 0.829, respectively). In the grassland habitat, the number of butterfly species and individuals exhibits a significant positive correlation with the variety of flowers (correlation coefficients: 0.857 and 0.845, respectively). Additionally, the Simpson dominance index of the butterfly community demonstrates a significant positive correlation with flower density (correlation coefficient: 0.812). In the forest-edge shrub habitat, however, the correlation between the species diversity of butterfly communities and that of nectar-source plants is not pronounced (Figure 10). For data from the Spearman correlation analysis, see Supporting Information SC: Spearman Correlation Analysis Results of Different Habitats.

## 4. Discussion

Based on our research results, the butterfly community in the Bailong River source area presents the following distinctive ecological characteristics along the elevational and habitat gradients.

### 4.1. Community Characteristics of Butterflies in Typical Alpine Grasslands

The Bailong River headwaters represent the westernmost edge of the Qinling Mountains, where butterfly communities form a distinct alpine grassland assemblage differing markedly from those of the Xiaolongshan forest area in the mid-western Qinling Mountains. Nymphalidae dominated the local butterfly fauna (60.0% of total species), followed by Pieridae (23.7%). Both proportions exceed values reported for Xiaolongshan forests (Nymphalidae: 55.6%; Pieridae: 15.38%), supporting Hypothesis (1): The species composition exhibits typical characteristics of alpine grasslands, with Nymphalidae and Pieridae being the most abundant families, while Papilionidae and Hesperiidae are the least abundant;. The open terrain of alpine grasslands favors long-distance flight and dispersal of Nymphalidae, while abundant Poaceae and Fabaceae provide sufficient food resources for Pieridae. In contrast, Papilionidae (0.7%) and Hesperiidae (0.5%) accounted for very low proportions, far below the 8.8% and 5.9% recorded in Xiaolongshan forests. This discrepancy arises from habitat preferences: Papilionidae and Hesperiidae rely mainly on warm, humid, low-elevation temperate broad-leaved forests with diverse host plants, habitats scarce within these high-altitude alpine valleys [34]. Lycaenidae maintained relatively stable representation across the two regions (13.0% at Bailong River headwaters vs. 15.7% in Xiaolongshan [8]), reflecting strong adaptability to both montane forest and alpine grassland habitats.

### 4.2. Pronounced Edge Effects at the Ecotone

Forest-edge shrub habitats had significantly higher Margalef richness and Shannon–Wiener diversity indices than alpine meadows and pure grasslands, consistent with Hypothesis (2);The ecotone generated pronounced habitat edge effects; the forest-edge shrub habitat harbored the highest species richness. No significant diversity differences were detected between alpine meadows and pure grasslands, confirming strong positive edge effects within this forest–grassland ecotone [21,22,25]. As transitional zones between forest and grassland ecosystems, forest-edge shrublands feature complex plant communities, vertical stratification, and high microhabitat heterogeneity. These sites combine the high plant diversity of forest interiors with the open space of grasslands, increasing the availability of larval host plants and adult nectar resources [25,35] and supporting richer butterfly assemblages. By comparison, pure grasslands and alpine meadows possess simpler vegetation and limited host-plant supplies and cannot sustain high butterfly diversity. In addition, forest-edge shrublands occur at lower elevations with warmer microclimates, offering better thermal conditions for butterfly activity relative to high-elevation alpine meadows [36].

### 4.3. Species Similarity Across Habitat Types

Butterfly assemblages exhibited clear habitat-specific clustering and limited species overlap among forest-edge shrubland, grassland, and alpine meadow habitats. This community differentiation stems largely from tight butterfly–host plant dependencies across insect life cycles. Most butterflies show strong host-plant specificity, and plants provide essential larval food and oviposition sites [8,37]. Previous work demonstrates that nectar plant diversity and abundance directly shape population dynamics, movement and spatial distributions of *Parnassius* butterflies [8,38,39]. Alpine meadows with high connectivity and dense flowering resources attract many migratory individuals [8,38,39]. Accordingly, habitat quality and vegetation structure are core drivers of alpine butterfly distribution and movement [40]. Variation in vegetation composition and habitat structure therefore generates pronounced community divergence among habitat types in the Bailong River headwaters.

### 4.4. A Distinct Double-Valley Pattern in the Elevational Distribution

PERMANOVA showed that butterfly species richness gradually declined with rising elevation, consistent with published studies [41,42,43,44] and supporting Hypothesis (3): butterfly species richness exhibits a monotonically decreasing trend with increasing altitude. Two non-mutually exclusive mechanisms may explain this elevational trend: the area hypothesis and the temperature-energy hypothesis. The area hypothesis states that high-elevation zones occupy smaller land areas, sustaining fewer individuals and fewer species toward mountain summits [45]. The temperature-energy hypothesis highlights thermal constraints: cooling temperatures and reduced available energy at higher elevations lower primary productivity, resource supply and carrying capacity for ectothermic animals such as butterflies, reducing abundance and species richness at high elevations [46].

One-way ANOVA further revealed that Margalef richness, Shannon diversity, and Simpson dominance indices generally decreased with increasing altitude. Two pronounced diversity valleys occurred at 2800–3000 m and 3200–3400 m. These sharp declines cannot be fully explained by area- or energy-driven background gradients, indicating additional negative impacts from human disturbance. The 2800–3000 m zone is a major tourism development area; intensive recreational activity and habitat modification cause severe disturbance and vegetation fragmentation, confirming strong effects of anthropogenic pressure on butterfly communities [27,47]. The 3200–3400 m zone is dominated by alpine grasslands experiencing long-term overgrazing. Both tourism and overgrazing degrade vegetation structure, reduce host and nectar plant diversity and abundance, and create local drops in butterfly diversity, forming this unique double-valley elevational pattern [27,47].

### 4.5. Conservation Implications

Butterfly communities in the Bailong River source area are mainly threatened by grassland degradation caused by long-term overgrazing and habitat deterioration induced by intensive tourism development. In addition, ongoing climate change poses severe survival threats to high-altitude Parnassiinae species, which are the most vulnerable insect groups and face potential local extinction risks [27,46]. Therefore, targeted butterfly conservation in this alpine watershed requires coordinated ecological management that balances socioeconomic development and biodiversity protection. Sustainable regional development and effective insect conservation can be achieved through harmonious integration of habitat restoration, ecological supervision, and moderate tourism and animal husbandry development.

Notably, alpine meadows above 3800 m represent the uppermost elevational distribution limit of local butterfly assemblages. Under continuous climate warming, high-altitude *Parnassius* butterflies have no available higher-elevation habitats for upward migration, resulting in extremely limited adaptive capacity to cope with climatic changes. Range-restricted mountain and polar insect species are particularly susceptible to severe population decline and range contraction under climate perturbation [27,45,48,49]. Accordingly, these endemic high-altitude butterfly species should be regarded as priority conservation targets for future biodiversity management in the Bailong River source area.

As an important ecological ecotone with rich biodiversity and pronounced edge effects, the headwaters of the Bailong River are ecologically valuable. Long-term ecological monitoring is therefore essential in this region [50]. We have established a systematic monitoring network comprising 50 fixed transects, of which 34 were surveyed in the present study, in Lixian and Gannan Prefecture.Prefecture, Gansu Province, China. Continuous field investigations have been conducted for five consecutive years from 2016 to 2022–2025, resulting in a long-term dataset of butterfly communities. Sustained monitoring in the future will provide fundamental support for the conservation and ecological research of mountain butterfly diversity.

## Figures and Tables

**Table 1:** Butterfly composition in the source area of the Bailong River.

Family	Individuals	Species	Genera	Proportion (%)
Nymphalidae	5577	57	33	60
Pieridae	2154	31	9	23.2
Lycaenidae	1457	32	19	15.7
Papilionidae	69	7	2	0.7
Hesperiidae	47	7	7	0.5

**Table 2:** Variations in the structure and diversity of butterfly communities across distinct habitats in the source region of the Bailong River.

Habitat Type	No. Species	Abundance	Shannon	Simpson	Pielou	Margalef
Grassland	42	619	2.7240	0.8841	0.3630	6.3780
Alpine meadow	41	1070	2.7890	0.9068	0.3966	5.7340
Forest–shrubland	105	3934	3.4150	0.9319	0.2896	12.5600

## Data Availability

The data presented in this study are available upon request from the corresponding author.

## Supplementary Materials

The following supporting information can be downloaded at: https://www.mdpi.com/article/10.3390/insects17090965/s1, Supporting Information SA: Environmental factors, and transect counting results. Supporting Information SB: Transects counting data. Supporting Information SC: Spearman Correlation Analysis Results of Different Habitats.
