# Supplementary material for: Impact of Habitat Changes on Butterfly Diversity in the Forest–Grassland Ecotone in the Source of the Bailong River, Western China
Source: Insects. 2026 Sep 19;17(9):965. doi: 10.3390/insects17090965 (PMC13607105; doi:10.3390/insects17090965)
Supplement: Supplementary file 1 [file insects-17-00965-s001.zip › Supporting Information SC_Spearman Correlation Analysis Results of Different Habitats.pdf]

Spearman Correlation Analysis Results of Different Habitats

S1. Alpine Meadow

Correlation (Spearman Rho)

Variable	Index	x1	x2	x3	x4	x5	x6	x7	x8
x1	Correlation Coefficient	1	.943**	.943**	.886*	.886*	-0.6	0.771	0.714
	Sig. (2-tailed)	.	0.005	0.005	0.019	0.019	0.208	0.072	0.111
	N	6	6	6	6	6	6	6	6
x2	Correlation Coefficient	.943**	1	.829*	0.714	0.714	-0.771	.886*	.829*
	Sig. (2-tailed)	0.005	.	0.042	0.111	0.111	0.072	0.019	0.042
	N	6	6	6	6	6	6	6	6
x3	Correlation Coefficient	.943**	.829*	1	.943**	.943**	-0.371	0.714	0.543
	Sig. (2-tailed)	0.005	0.042	.	0.005	0.005	0.468	0.111	0.266

Variable	Index	x1	x2	x3	x4	x5	x6	x7	x8
	N	6	6	6	6	6	6	6	6
x4	Correlation Coefficient	.886*	0.714	.943**	1	1.000**	-0.257	0.486	0.371
	Sig. (2-tailed)	0.019	0.111	0.005	.	.	0.623	0.329	0.468
	N	6	6	6	6	6	6	6	6
x5	Correlation Coefficient	.886*	0.714	.943**	1.000**	1	-0.257	0.486	0.371
	Sig. (2-tailed)	0.019	0.111	0.005	.	.	0.623	0.329	0.468
	N	6	6	6	6	6	6	6	6
x6	Correlation Coefficient	-0.6	-0.771	-0.371	-0.257	-0.257	1	-0.6	-0.6
	Sig. (2-tailed)	0.208	0.072	0.468	0.623	0.623	.	0.208	0.208
	N	6	6	6	6	6	6	6	6

Variable	Index	x1	x2	x3	x4	x5	x6	x7	x8
x7	Correlation Coefficient	0.771	.886*	0.714	0.486	0.486	-0.6	1	.886*
	Sig. (2-tailed)	0.072	0.019	0.111	0.329	0.329	0.208	.	0.019
	N	6	6	6	6	6	6	6	6
x8	Correlation Coefficient	0.714	.829*	0.543	0.371	0.371	-0.6	.886*	1
	Sig. (2-tailed)	0.111	0.042	0.266	0.468	0.468	0.208	0.019	.
	N	6	6	6	6	6	6	6	6

Note: ** Correlation is significant at the 0.01 level (2-tailed). * Correlation is significant at the 0.05 level (2-tailed).

S2. Grassland

Correlation (Spearman Rho)

Variable	Index	x1	x2	x3	x4	x5	x6	x7	x8
x1	Correlation Coefficient	1	.928**	.928**	.928**	0.638	-0.696	.857*	0.338

Variable	Index	x1	x2	x3	x4	x5	x6	x7	x8
	Sig. (2-tailed)	.	0.008	0.008	0.008	0.173	0.125	0.029	0.512
	N	6	6	6	6	6	6	6	6
x2	Correlation Coefficient	.928**	1	0.771	0.771	0.371	-.886*	.845*	-0.029
	Sig. (2-tailed)	0.008	.	0.072	0.072	0.468	0.019	0.034	0.957
	N	6	6	6	6	6	6	6	6
x3	Correlation Coefficient	.928**	0.771	1	1.000**	.829*	-0.429	0.676	0.522
	Sig. (2-tailed)	0.008	0.072	.	.	0.042	0.397	0.140	0.288
	N	6	6	6	6	6	6	6	6
x4	Correlation Coefficient	.928**	0.771	1.000**	1	.829*	-0.429	0.676	0.522
	Sig. (2-tailed)	0.008	0.072	.	.	0.042	0.397	0.140	0.288

Variable	Index	x1	x2	x3	x4	x5	x6	x7	x8
	N	6	6	6	6	6	6	6	6
x5	Correlation Coefficient	0.638	0.371	.829*	.829*	1	0.086	0.507	.812*
	Sig. (2-tailed)	0.173	0.468	0.042	0.042	.	0.872	0.305	0.050
	N	6	6	6	6	6	6	6	6
x6	Correlation Coefficient	-0.696	-.886*	-0.429	-0.429	0.086	1	-0.676	0.377
	Sig. (2-tailed)	0.125	0.019	0.397	0.397	0.872	.	0.140	0.461
	N	6	6	6	6	6	6	6	6
x7	Correlation Coefficient	.857*	.845*	0.676	0.676	0.507	-0.676	1	0.257
	Sig. (2-tailed)	0.029	0.034	0.140	0.140	0.305	0.140	.	0.623
	N	6	6	6	6	6	6	6	6

Variable	Index	x1	x2	x3	x4	x5	x6	x7	x8
x8	Correlation Coefficient	0.338	-0.029	0.522	0.522	.812*	0.377	0.257	1
	Sig. (2-tailed)	0.512	0.957	0.288	0.288	0.050	0.461	0.623	.
	N	6	6	6	6	6	6	6	6

Note: ** Correlation is significant at the 0.01 level (2-tailed). * Correlation is significant at the 0.05 level (2-tailed).

S3. Forest Edge Shrubland

Correlation (Spearman Rho)

Variable	Index	x1	x2	x3	x4	x5	x6	x7	x8
x1	Correlation Coefficient	1	0.493	0.551	0.493	0.464	0.058	-0.279	-0.522
	Sig. (2-tailed)	.	0.321	0.257	0.321	0.354	0.913	0.592	0.288
	N	6	6	6	6	6	6	6	6
x2	Correlation Coefficient	0.493	1	-0.086	-0.486	-0.143	-0.543	-0.696	-.886*
	Sig. (2-	0.32	.	0.872	0.32	0.787	0.26	0.12	0.019

Variable	Index	x1	x2	x3	x4	x5	x6	x7	x8
	tailed)	1			9		6	5	
	N	6	6	6	6	6	6	6	6
x3	Correlation Coefficient	0.551	-0.086	1	0.657	.943**	0.771	0.058	-0.029
	Sig. (2-tailed)	0.257	0.872	.	0.156	0.005	0.072	0.913	0.957
	N	6	6	6	6	6	6	6	6
x4	Correlation Coefficient	0.493	-0.486	0.657	1	0.6	0.6	0.319	0.314
	Sig. (2-tailed)	0.321	0.329	0.156	.	0.208	0.208	0.538	0.544
	N	6	6	6	6	6	6	6	6
x5	Correlation Coefficient	0.464	-0.143	.943**	0.6	1	.886*	-0.058	-0.086
	Sig. (2-tailed)	0.354	0.787	0.005	0.208	.	0.019	0.913	0.872
	N	6	6	6	6	6	6	6	6

Variable	Index	x1	x2	x3	x4	x5	x6	x7	x8
x6	Correlation Coefficient	0.058	-0.543	0.771	0.6	.886*	1	0.145	0.257
	Sig. (2-tailed)	0.913	0.266	0.072	0.208	0.019	.	0.784	0.623
	N	6	6	6	6	6	6	6	6
x7	Correlation Coefficient	-0.279	-0.696	0.058	0.319	-0.058	0.145	1	.928**
	Sig. (2-tailed)	0.592	0.125	0.913	0.538	0.913	0.784	.	0.008
	N	6							
